# Relationship between aeroallergen sensitization pattern and clinical features in adult asthmatics

**DOI:** 10.1016/j.heliyon.2023.e15708

**Published:** 2023-04-25

**Authors:** Dilek Karadoğan, Tahsin Gökhan Telatar, Halil Dönmez, Adile Berna Dursun

**Affiliations:** a Department of Chest Diseases, Recep Tayyip Erdoğan University, School of Medicine, Rize, Turkey; b Department of Public Health, Recep Tayyip Erdoğan University, School of Medicine, Rize, Turkey; c Department of Immunology and Allergic Diseases, Recep Tayyip Erdoğan University, Rize, Turkey; d Department of Chest Diseases, Lokman Hekim University, School of Medicine, Ankara, Turkey

**Keywords:** Asthma, Aeroallergen sensitivity, Non-atopic, Atopic, Asthma control, Asthma severity

## Abstract

**Background:**

Asthma can be classified into atopic and non-atopic phenotypes. However, limited data are available on the clinical implications of these two phenotypes in real life.

**Objective:**

This study aimed to examine the clinical features as well as control level and disease severity of asthmatic patients with their aeroallergen sensitivity profiles.

**Methods:**

Between 2013 and 2020, adult asthmatic patients who had been followed up regularly at our tertiary healthcare institution for at least one year were included in the study. We collected data retrospectively using manually filled patient files.

**Results:**

The mean age of 382 asthmatic patients was 46.6 ± 30.0; 77.5% were women and 75.6% had at least one aeroallergen sensitivity. Polysensitized asthmatics had better asthma symptom control and milder asthma severity than monosensitized asthmatics. Asthma symptom control status was well controlled in 67.5% of the patients, and according to asthma severity, 51.3% of the patients were classified as having moderate asthma. There was a negative relation between age (OR:0.95, CI:0.92–0.98) and atopy presence. The presence of atopy was higher in moderate asthmatics than in mild asthmatics (OR:2.02, CI:1.01–4.09). Finally, there was a positive relationship between the percent predicted forced expiratory volume in first second (FEV_1_%) (OR:1.02, CI:1.009–1.048) and the presence of atopy. The presence of rhinitis (OR:0.44, CI:0.22–0.88) and per 1 unit increase of Tiffeneau index (FEV_1_/forced vital capacity) (OR:0.94, CI:0.90–0.99) had a negative association, whereas number of medication use for asthma symptoms (OR:1.68, CI:1.18–2.39) and presence of cardiovasculary disease (OR:2.64, CI:1.19–5.84) had a positive association with not well-controlled asthma symptom level.

**Conclusion:**

Aeroallergen sensitivity was associated with asthma severity. However, this was not the case with asthma control levels in this adult asthma cohort. Among the atopic asthmatics polysensitized asthmatics had better asthma symptom control level and milder asthma severity level.

## 1 Introduction

The prevalence of asthma is increasing because of air pollution and occupational exposure [1]. Asthma is generally classified as either atopic or non-atopic. Although approximately 60% of adult asthmatics suffer from an atopic phenotype [2,3], few studies have compared the differences between atopic and non-atopic asthma. The findings of these studies are inconsistent. While some studies have established a relationship between atopic asthma and poorer clinical outcomes [4], others have found no such difference [5,6]. The impact of atopy has not been adequately evaluated regarding the morbidity, control level, and severity of asthma.

In a multicenter study from Turkey, a total of 1400 asthmatic patients were evaluated at 14 centers across Turkey; of those, 36.3% had allergic asthma, with 289 patients being monosensitive and 182 polysensitive. Mite, grass pollen mix, and molds are the most commonly observed aeroallergens [7]. A recent study in Turkey indicated that mites, cockroaches, and grass pollen are the most common aeroallergens among allergic rhinitis and asthmatic patients [8]. Moreover, the patients who were sensitive to olive pollen were described as having mild asthma, while those who were sensitive to mites were classified as having moderate to severe asthma. In our previous study that aimed to evaluate the clinical characteristics of asthma in terms of mold sensitivity status revealed the association between the mold sensitivity and well-controlled asthma symptom level [9]. Conversely, some studies have demonstrated that poor asthma control depends on the geographic characteristics of the setting and sample [10,11]. Aeroallergen sensitivity status is being evaluated by using the skin prick test and specific measurements of IgE levels andincreased IgE levels were associated with worse outcomes in atopic asthmatics, whereas no such association was found in non-atopic asthmatics [10]. Additionally, a recentstudy investigated the effects of smoking on adult-onset asthma regarding gender, highlighting the effects of smoking on both atopic and non-atopic onset asthma [11].

Due to the above pointed controversial knowledge gap in the literature on this subject, this study aimed to evaluate the relation between aeroallergen sensitization patterns and clinical outcomes of asthmatic patients who were followed regularly in the Eastern Black Sea Region of Turkey, which has a humid subtropical climate due to year-round rainfall [12].

## 2 Materials and methods

### 2.1 Study design

This retrospective study was designed at the Department of Adult Allergic Diseases Clinic in the Eastern Black Sea region of Turkey. All asthma-diagnosed patients were evaluated between June 2013 and September 2020. Additionally, this study used the follow-up files as the source material for only those patients whose files were routinely updated by the allergy fellows.

Inclusion criteria were as follows.

1. Patients that met the diagnosis criterias for asthma according to Global Initiative for Asthma guideline (GINA) [13].
2. Asthmatic patients with at least one year of regular follow-up at the clinic for allergic diseases
3. Asthmatic patients aged ≥18 years.

Patients with missing data or less than 1 year follow-up were excluded. The institutional ethics committee approved the study protocol on August 20, 2020 with the number 2020/180.

### 2.2 Data collection

We obtained demographic information—age, sex, education level, comorbidities, asthma clinical history, and skin prick test (SPT) results—from manually filled patient files at their initial visits. In addition, we evaluated asthma symptom control tests, asthma severity levels, and pulmonary function tests (PFTs) from their first-year follow-up records.

For each patient, an SPT was administered involving common aeroallergens, including *Dermatophagoides pteronyssinus* and *Dermatophagoides farinae* (dust mites); *Aspergillus*, *Alternaria*, *Cladosporium,* and *Penicillium* (molds); cat and dog dander; latex; pollens from grass, trees, and weeds; and cockroaches (Allergopharma, Reinbek, Germany). SPTs were administered on the volar forearm and interpreted after 20 min. A wheal reaction with a mean diameter of 3 mm greater than that of the negative control was considered positive.

Recorded PFTs had been performed according to the recommendations [14] using a pulmonary spirometer (CareFusion, Germany, 234 GmbH). Post-bronchodilator forced expiratory volume in the first second (FEV_1_), forced vital capacity (FVC), and forced expiratory flow at 25–75% of FVC (FEF_25–75_) values were obtained from the study files of the participants. Our SPT and PFT procedures have been described in detail in the Methods section of our previous studies [9,15]. The GINA assessment of asthma symptom control questionnaire in adults was used to evaluate asthma symptom control level. Asthma severity was assessed retrospectively based on the level of treatment required to control symptoms and exacerbations, as recommended by the GINA [16].

### 2.3 Statistical analysis

The data were analyzed using the SPSS 22.0 package program. Categorical variables are presented as numbers and percentages, and continuous variables as means and standard deviations if normally distributed and medians and interquartile ranges (IQR) if otherwise. The distribution properties of continuous variables were evaluated using the Kolmogorov-Smirnov test, the chi-square test, Fisher's exact test, students-t test and Mann Whitney *U* test to determine the relations between sociodemographic characteristics and clinical features of participants' aeroallergen sensitivities. The effects of statistically significant variables, that was analyzed by univariate analysis, on the presence of atopy and their effects on asthma symptom control level were evaluated using a logistic regression model.

## 3 Results

The mean age of the 382 patients was 46,6 ± 30,0, with 77.5% female and 22.5% male. Most patients had never smoked (80.1%), 10.2% were current smokers, 54% of the patients had obesity according to BMI and 76.4% suffered from rhinitis. According to the asthma symptom control level, 67.5% of the patients had well-controlled, with 19.1% and 13.4% having partly controlled and uncontrolled asthma symptom levels, respectively. Regarding asthma severity, 51.3% of the patients had moderate asthma, whereas 41.4% had mild asthma, and 7.3% had severe asthma (Table 1).

**Table 1:** Comparison of clinical variables of asthma patients according to their aeroallergen sensitization patterns.

	Total (n:382)	Non-atopic (n:93)	Atopic (n:289)	p
**Age,** mean ± SD	46.61 ± 30.04	54.83 ± 14.22	43.9 ± 14.8	<0.001
**Sex,** n (%)				0.15
Female	296 (77.5)	77 (82.7)	219 (75.7)	
Male	86 (22.5)	16 (17.2)	70 (24.2)	
**Smoking status, n (%)**				0.43
Never smoker	306 (80.1)	76 (81.7)	230 (79.5)	
Current smoker	39 (10.2)	11 (11.8)	28 (9.6)	
Former smoker	37 (9.7)	6 (6.4)	31 (10.7)	
**BMI,** median (IQR)	29.6 (7.7)	31.0 (8.49)	29.0 (7.09)	0.011
**BMI group,** n (%)				0.02
≥30 kg/m^2^	165 (46.0)	49 (52.6)	116 (40.1)	
<30 kg/m^2^	194 (54.0)	38 (40.8)	156 (53.9)	
**Presence of rhinitis,** n (%)	292 (76.4)	57 (61.2)	235 (81.3)	<0.001
**Presence of sinonasal polyposis,** n (%)	50 (13.1)	20 (21.5)	30 (10.3)	0.006
**Presence of chronic rhinosinusitis,** n (%)	6 (1.6)	0 (0)	6 (2.07)	0.16
**Presence of other comorbid diseases,** n (%)	144 (37.7)	52 (55.9)	92 (31.8)	<0.001
**Presence of metabolic diseases,** n (%)	58 (15.2)	20 (21.5)	38 (13.1)	0.051
**Presence of cardiovascular disease,** n (%)	79 (20.7)	36 (38.7)	43 (14.8)	<0.001
**Presence of psychiatric disease,** n (%)	22 (5.8)	10 (10.7)	12 (4.15)	0.017
**Presence of gastroesophageal reflux disease,** n (%)	15 (3.9)	2 (2.15)	13 (4.49)	0.31
**Asthma initiation age,** median (IQR)	38.0 (21.0)	45.0 (19.0)	35.0 (21.0)	<0.001
**Asthma duration,** median (IQR)	7.0 (10.0)	7.0 (13.0)	6.0 (10.0)	0.767
**Level of asthma symptom control,** n (%)				0.137
Well-controlled	258 (67.5)	55 (59.1)	203 (70.2)	
Partly controlled	73 (19.1)	22 (23.6)	51 (17.6)	
Uncontrolled	51 (13.4)	16 (17.2)	35 (12.1)	
**Asthma severity,** n (%)				0.415
Mild asthma	158 (41.4)	41 (44.0)	117 (40.4)	
Moderate asthma	196 (51.3)	43 (46.2)	153 (52.9)	
Severe asthma	28 (7.3)	9 (9.6)	19 (6.57)	
**Used medication number for asthma related symptoms,** median (IQR)	2.00 (1.00)	2.0 (1.0)	2.0 (1.0)	0.016
**Lifetime hospitalization number,** median (IQR)	0.0 (0.0)	0.0 (1.0)	0 (0)	0.004
**Lifetime emergency department visit number,** median (IQR)	0.0 (3.0)	1 (4)	0 (2)	0.161
**FEV**_**1**_**(L),** median (IQR)	2.59 (1.17)	2.22 (0.93)	2.72 (1.75)	<0.001
**FEV**_**1**_**(%),** median (IQR)	94.0 (19.0)	90.0 (16.7)	95.5 (18.0)	0.002
**FEV**_**1**_**/FVC,** median (IQR)	77.6 (10.3)	77.5 (13.6)	77.6 (10.2)	0.611
**FEF**_**25-75**_**(L),** median (IQR)	2.36 (1.73)	2.06 (1.90)	2.55 (1.68)	0.001
**FEF**_**25-75**_**(%),** median (IQR)	70.0 (43.2)	65.5 (49.5)	70.5 (41.2)	0.149

Abbrevations: BMI: Body mass index, IQR: Interquartile range, FEV_1_: Forced expiratory volume in first second, FVC: Forced vital capacity, FEF_25-75_: The forced mid-expiratory flow, L: Liter.

**Fig. 1:**
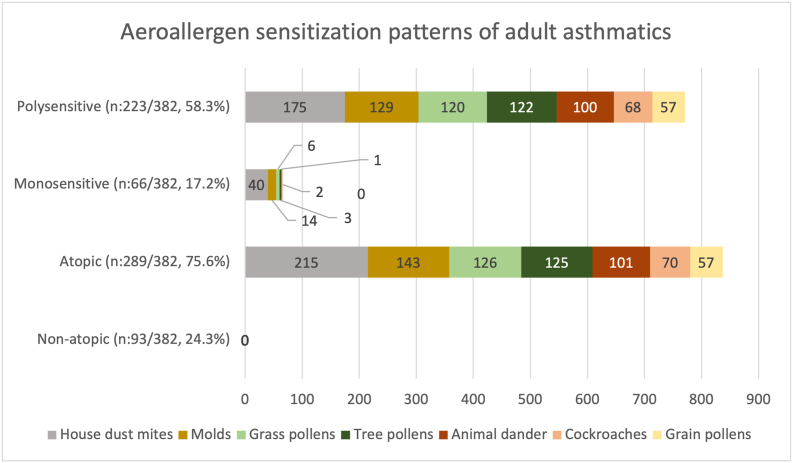
Aeroallergen sensitization patterns of adult asthmatics.

According to the skin prick test results, 223 patients (58.3%) had polysensitization, while 66 (17.2%) had monosensitization, with the rest of 93 (24.3%) having negative SPT results. According to the SPT, the aeroallergen sensitivity status and the frequency were as follows: mites (56.2%), molds (37.4%), grass pollen (32.9%), tree pollen (32.7%), animal dander (26.4%), cockroaches (18.3%), and grain pollen (14.9%) (Fig. 1).

Table 2 shows the comparison of atopic asthmatics regarding their sensitization patterns. Compared to monosensitized asthmatics, polysensitized group's mean age, median asthma onset age and median asthma duration were lower. Rates of asthmatics with well controlled symptom level and mild asthmatics were higher in polysensitized asthmatics than monosensitized counterparts.

**Table 2:** Comparison of clinical variables of atopic asthmatics according to their aeroallergen sensitization patterns.

	Monosensitive (n: 66)	Polysensitive (n: 223)	p
**Age,** mean ± SD	50.68 ± 14.95	41.98 ± 14.3	<0,001
**Sinonasal polyposis,** n (%)	13 (19.6)	17 (7.6)	0,005
**Presence of other comorbid diseases,** n (%)	31 (46.9)	61 (27.3)	0,003
**Presence of cardiovascular disease,** n (%)	20 (30.3)	23 (10.3)	<0,001
**Asthma onset age,** median (IQR)	41.0 (25.0)	34.0 (22.0)	0,008
**Asthma duration,** median (IQR)	10.0 (13.0)	6.0 (9.0)	0,025
**Level of asthma symptom control,** n (%)			0,023
Well-controlled	39 (59.0)	164 (73.5)	
Partly controlled	19 (28.7)	32 (14.3)	
Uncontrolled	8 (12.1)	27 (12.1)	
**Asthma severity,** n (%)			0,014
Mild asthma	17 (25.7)	100 (44.8)	
Moderate asthma	42 (63.6)	111 (49.7)	
Severe asthma	7 (10.6)	12 (5.38)	
**Lifetime emergency department visit number,** n (%)			0,032
5 and lower	51 (77.2)	196 (87.8)	
6 and over	15 (22.7)	27 (12.1)	
**FEV**_**1**_**(L),** median (IQR)	2.33 (0.88)	2.80 (1.16)	0.001
**FEF**_**25-75**_**(L),** median (IQR)	2.16 (1.35)	2.67 (1.89)	0,007

Abbrevations: BMI: Body mass index, IQR: Interquartile range, **FEV**_**1**_**:** Forced expiratory volüme in first second, FVC: Forced vital capacity, **FEF**_**25-75**_**:** The forced mid-expiratory flow, L: Liter.

Table 3 shows the multivariate logistic regression model of variables detected to have statistically significant effect on presence of atopy in the univariate analysis that is also seen in Table 1. Per 1 age increase was negatively associated with atopy presence (OR:0.95, CI:0.92–0.98). The presence of atopy was higher in moderate asthmatics than in mild asthmatics (OR:2.02, CI:1.01–4.09). Per 1 unit increase in FEV_1_% predicted was positively associated with presence of atopy (OR:1.02, CI:1.009–1.048).

**Table 3:** Factors associated with presence of atopy in multivariate logistic regression analysis.

	Odds Ratio	p	95% CI (lower -upper)
**Age** (per 1 age increase)	0.953	0.005*	0.921–0.985
**Sex**			
Female	REF		
Male	0.888	0.777	0.392–2.012
**BMI**			
<30 kg/m^2^	REF		
≥30 kg/m^2^	1.138	0.665	0.634–2.042
**Sinonasal polyposis**			
Absent	REF		
Present	0.598	0.175	0.285–1.256
**Asthma initiation age** (per 1 age increase)	0.988	0.424	0.959–1.018
**Asthma severity**			
Mild	REF		
Moderate	2.053	0.044*	1.01 9–4.136
Severe	1.928	0.332	0.512–7.266
**FEV**_**1**_**% predicted** (per 1 unit increase)	1.028	0.004*	1.009–1.048
**FEV**_**1**_**/FVC** (per 1 unit increase)	0.957	0.075	0.911–1.004
**FEV**_**25–75**_**%** (per 1 unit increase)	1.099	0.681	0.702–1.719
**Level of asthma symptom control**			
Well-controlled	REF		
Partly-controlled	0.765	0.515	0.342–1.713
Uncontrolled	0.683	0.461	0.249–1.879
**Constant**	**75.449**	**0.015**	

Abbrevations: BMI: Body mass index, FEV_1_: Forced expiratory volüme in first second, FVC: Forced vital capacity, FEF_25-75_: The forced mid-expiratory flow, REF: Reference category.

Table 4 shows the multivariate logistic regression model of variables detected to have statistically significant effect regarding asthma control level in univariate analysis. Presence of rhinitis (OR:0.44, CI:0.22–0.88) and per 1 unit increase in FEV_1_/FVC (OR:0.94, CI:0.90–0.99) were negatively associated with not well controlled asthma symptoms. Presence of CVD (OR:2.64, CI:1.19–5.84) and per 1 medication number, used for asthma related symptoms, increase (OR:1.68, CI:1.18–2.39) negatively associated with not well-controlled asthma symptoms.

**Table 4:** Factors associated with not well-controlled (partly controlled or uncontrolled) asthma symptom level.

	Odds Ratio	p	95% CI (lower -upper)
**Age** (per 1 year increase)	2.087	0.142	0.78–5.57
**BMI**			
<30 kg/m^2^	REF		
≥30 kg/m^2^	1.240	0.440	0.71–2.14
**Asthma initiation age** (per 1 age increase)	0.479	0.142	0.17–1.28
**Asthma duration** (per 1 year increase)	0.487	0,152	0.18–1.30
**Rhinitis**			
Absent	REF		
Present	0.44	0.019*	0.22–0.88
**Other comorbid diseases**			
Absent	REF		
Present	1.46	0.317	0.69–3.08
**Metabolic diseases**			
Absent	REF		
Present	1.48	.0319	0.67–3.26
**Cardiovascular diseases**			
Absent	REF		
Present	2.64	0.016*	1.19–5.84
**Sensitivity to tree pollens**			
Absent	REF		
Present	0.61	0.180	0.32–1.23
**Sensitivity to grain pollens**			
Absent	REF		
Present	0.98	0.980	0.42–2.28
**Sensitization patterns in skin prick test**			
Non-atopic asthmatics	REF		
Monosensitized asthmatics	1.36	0.435	0.62–2.97
Polysensitized asthmatics	1.30	0.495	0.60–2.79
**Medication number** (per 1 number increase)	1.68	0.003*	1.18–2.39
**FEV**_**1**_**% predicted** (per 1% increase)	0.98	0.178	0.96–1.01
**FEV**_**1**_**/FVC** (per 1 unit increase)	0.94	0.035*	0.90–0.99
**FEV**_**25-75**_ (per 1 unit increase)	0.81	0.482	0.45–1.44
**FEF**_**25–75**_**%** (per 1% increase)	1.02	0.121	0.99–1.04
**Constant**	**0.229**	**10.49**	

Abbrevations: BMI: Body mass index, FEV_1_: Forced expiratory volüme in first second, FVC: Forced vital capacity, FEF_25-75_: The forced mid-expiratory flow, REF: Reference category.

## 4 Discussion

Our results evaluated the adult asthmatics according to their aeroallergen sensitivity status, and compared to non-atopics, atopic asthmatics were younger, have higher FEV_1_% predicted and have higher rate of moderate asthmatics. Among atopic asthmatics majority had polysensitivity and polysensitized asthmatics had better asthma symptom control level and milder asthma severity compared to monosensitized asthmatics. Presence of rhinitis and higher FEV_1_/FVC were positively associated with well asthma symptom control, whereas presence of CVD and higher medication number were negatively associated with well controlled asthma symptoms.

Regarding aeroallergen sensitivity, 25% were non-atopic, the majority (57%) were polysensitive, and the remaining 18% were monosensitive. The most frequently detected aeroallergens were house dust mites (HDM), molds, and pollen. Our findings are consistent with those of national and international studies on adult asthmatic patients [8,11,17,18]. Studies have reported that levels of skin prick test reactivity vary with changes in climate, humidity, and meteorological factors [19,20]. In a study conducted in the Marmara region of Turkey, 148 of 280 (52%) asthmatic patients exhibited SPT sensitivity. The most common aeroallergen was HDM, followed by pollen and cockroaches; however, this frequency varied based on the presence of other allergic diseases accompanying asthma [21]. Another study examined the patterns of aeroallergen sensitivity in 749 asthmatic patients in Mexico who were evaluated using the SPT. The prevalence of sensitization was 77% in the adult asthmatic group [18]. Based on aeroallergen sensitivity, 63% of asthmatics were found to be atopic in another study [22]. Owing to the rainy climate, the high humidity level of mold sensitivity rate is the second most commonly discovered aeroallergen in our study population [9]. A previous study that included various regions of Turkey demonstrated that the presence of mites was associated with an increase in both mean temperatures (>15 °C), high relative humidity (340%), and low altitude (<300 m) [23]. Located at sea level, the eastern Black Sea region has high humidity from heavy rains throughout the year [12]. In a recent study, an alpine altitude climate was found to be associated with good asthma control owing to reduced aeroallergens and less air pollution [24].

Comparisons regarding atopy status revealed differences and multivariate logistic regression model showed that; compared to non-atopics, atopic asthmatics were younger, have higher FEV_1_% predicted and have higher rate of moderate asthmatics. It was reported that non-atopic asthmatics were more often female, older, and less educated than those in atopic and control groups [20]. In another study also atopic asthmatics have been reported to have a greater incidence of early-onset asthma and higher mean FEV_1_, FVC, and FEV_1_/FVC values than non-atopic asthmatics [25]. Several studies have reported a relationship between asthma severity and atopy. The studies comparing atopic and non-atopic asthma discovered no significant differences in asthma control. However, other studies have observed differences between asthma severity and atopy status. A longitudinal study that followed patients for one year revealed that atopic asthmatics had more severe asthma than non-atopic asthmatics. Other studies have noticed that these findings are compatible with those of previous studies conducted on different populations. In other words, the severity of asthma was discovered to be greater in atopic asthmatics considering factors such as increased symptoms, increased drug use, and increased exacerbation [17]. In another study, the rate of non-atopic asthma was higher in patients with severe asthma [26]. According to our findings, among atopic asthmatics majority had polysensitivity and polysensitized asthmatics had better asthma symptom control and milder asthma severity compared to monosensitized asthmatics. However, we found no difference in asthma control or severity specific to any allergen type. Similarly, a study revealed no significant difference between atopic and non-atopic asthmatics based on their asthma control test scores [22]. In a recent study, the olive sensitivity rate was higher in patients with mild asthma, while the HDM sensitivity rate was higher in moderate asthmatics. However, no adjusted analysis was performed when confounders or effect modifiers were considered [8]. Our previous study revealed that mold sensitivity was positively associated with well-controlled asthma [23].

The effectors that have been reported to have role on poor asthma control such as advanced age, late onset asthma, obesity, comorbidities [27] were lower among our polysensitized asthmatics than monosensitized ones. Presence of rhinitis and higher FEV_1_/FVC were positively associated with well asthma symptom control, whereas presence of CVD and higher medication number were negatively associated with well controlled asthma symptoms. In the last decade, the percentage of patients with uncontrolled asthma has ranged from 45 to 57% [28]. A study examined the patterns of aeroallergen sensitivity in 749 asthmatic patients in Mexico and a 37% of the total patients were identified to be well controlled [18]. In European population-based studies, the prevalence of poorly controlled asthma ranged from 56% to 80%. Regarding asthma control levels, inconsistency between randomized controlled trials and intervention studies has also been reported due to the difference in the methodology applied. Moreover, the use of different definitions and measurements of control also leads to different results. Treatment-related conditions such as poor adherence, inadequate treatment, and inadequate inhalation techniques are the most important factors for poor asthma control [29]. Most of our patients had well-controlled asthma symptoms (67.5%), 19.1% had partly controlled asthma symptoms, and 13.4% had uncontrolled symptom levels. Another factor reported to be effective in asthma control is the qualifications of the treating physician. In a novel study overprescription of short-acting beta-2 agonists has been associated with poor asthma symptom control in Middle-East coutries and that was found to be underscoring the need for healthcare providers to adhere to the latest evidence-based recommendations to address this public health concern [30]. The follow-up of our patients by qualified physicians in the allergy clinic effectively ensured a high rate of good control. In addition, regular follow-ups, close monitoring of patient compliance, and training on disease and avoidance of triggers are necessary to achieve a high rate of good control.

## 5 Strenghts and limitations

The strength of the study is the completeness of the files that data was carefully filled by expert allergists. The retrospective study design limits the evaluation of the causal relationships. Data collection from one center instead of multicenter design also limits the generalization of the results to all population. Due to not having large sample size, grouping main types of aeroallergens instead of presenting detailed types of aeroallergen species such as kind of trees, kind of grains are also among the limitations.

In conclusion, all adult asthmatic patients who were followed up regularly in the immunology and allergic diseases clinic in a humid, rainy climate region were evaluated. According to the skin prick test results, aeroallergen sensitivity was discovered in 75% of asthmatic patients, with HDM, mold, and pollen being the most common allergens. Younger age, higher FEV_1_% predicted and presence of moderate asthma severity was associated with presence of atopy. Among atopic asthmatics polysensitized ones had better asthma symptom control level as well as higher rate of remission predictors for asthma compared to monosensitized counterparts.

## Author contribution statement

Dilek Karadoğan: Conceived and designed the experiments; Analyzed and interpreted the data; Contributed reagents, materials, analysis tools or data; Wrote the paper.

Tahsin Gökhan Telatar: Analyzed and interpreted the data; Contributed reagents, materials, analysis tools or data.

Halil Dönmez: Performed the experiments; Contributed reagents, materials, analysis tools or data.

Adile Berna Dursun: Conceived and designed the experiments; Performed the experiments; Analyzed and interpreted the data; Contributed reagents, materials, analysis tools or data; Wrote the paper.

## Data availability statement

Data will be made available on request.

## Additional information

Supplementary content related to this article has been published online at [URL].

## Declaration of interest's statement

The authors declare that they have no known competing financial interests or personal relationships that could have appeared to influence the work reported in this paper.

The authors declare no conflict of interest.
